# Inhibition of Proteasome Activity by Low-dose Bortezomib Attenuates Angiotensin II-induced Abdominal Aortic Aneurysm in Apo E^−/−^ Mice

**DOI:** 10.1038/srep15730

**Published:** 2015-10-28

**Authors:** Hualiang Ren, Fangda Li, Cui Tian, Hao Nie, Lei Wang, Hui-Hua Li, Yuehong Zheng

**Affiliations:** 1 Department of Vascular Surgery, Peking Union Medical College Hospital, Beijing, PR China; 2 Department of Physiology and Physiopathology, Beijing AnZhen Hospital the Key Laboratory of Remodeling-Related Cardiovascular Diseases, Beijing Key Laboratory of Cardiovascular Diseases Related to Metabolic Disturbance, School of Basic Medical Sciences, Capital Medical University, Beijing, PR China; 3 Department of Cardiology, Institute of Cardiovascular Diseases, First Affiliated Hospital of Dalian Medical University, Dalian 116011, PR China; 4 Center for Prevention and Control of Non-communicable Chronic Diseases, School of Public Health, Dalian Medical University, Dalian 116044, PR China

## Abstract

Abdominal aortic aneurysm (AAA) is a leading cause of sudden death in aged people. Activation of ubiquitin proteasome system (UPS) plays a critical role in the protein quality control and various diseases. However, the functional role of UPS in AAA formation remains unclear. In this study, we found that the proteasome activities and subunit expressions in AAA tissues from human and angiotensin II (Ang II)-infused apolipoprotein E knockout (Apo E^−/−^) mice were significantly increased. To investigate the effect of proteasome activation on the AAA formation, Apo E^−/−^ mice were cotreated with bortezomib (BTZ) (a proteasome inhibitor, 50 μg/kg, 2 times per week) and Ang II (1000 ng/kg/min) up to 28 days. Ang II infusion significantly increased the incidence and severity of AAA in Apo E^−/−^ mice, whereas BTZ treatment markedly inhibited proteasome activities and prevented AAA formation. Furthermore, BTZ treatment significantly reduced the inflammation, inhibited the metal matrix metalloprotease activity, and reversed the phenotypic SMC modulation in AAA tissue. In conclusion, these results provide a new evidence that proteasome activation plays a critical role in AAA formation through multiple mechanisms, and suggest that BTZ might be a novel therapeutic target for treatment of AAA formation.

Abdominal aortic aneurysm (AAA) is characterized by vascular remodeling, degradation of extracellular matrix (EMC), immune responses, cell apoptosis, and neovascularization of the media and adventitia^1^,^2^. AAA is a major cause of sudden death in aged people. Risk factors for AAA include age, male gender, genetic predisposition, atherosclerosis, and hypertension. Although many theories have been advanced to explain the development of AAA, the molecular mechanisms for AAA progression and effective drugs for conservative treatment have not been defined^3^. Among the numerous pathophysiological mechanisms, chronic aortic wall inflammation^4^, especially T lymphocyte and macrophage infiltration, plays a central role in AAA formation^5^. Cytokines are released by T cells and macrophages, including interferon-gamma (IFN-γ), transforming growth factor-beta (TGF-β), and osteopontin (OPN)^1^, which induce matrix metallo-proteinase activity, EMC degradation and smooth muscle cell (SMC) phenotype change leading to AAA formation^5^,^6^.

The ubiquitin–proteasome system (UPS) is the major pathway for cytosolic and nuclear protein degradation in eukaryotic cells, and regulates various cell proteins that are essential for regulating inflammation, phenotype change, signal transduction, and stress response^7^. The central proteolytic UPS unit is the 26S proteasome, a large multicatalytic multisubunit protease composed of a 20S barrel-shaped catalytic core complex and two flanking 19S regulatory complexes^8^. The proteolytic activities of the 26S proteasome occur in the 20S complex, composed of four axially stacked rings. Each outer ring contains seven different α-subunits, and each of the two inner rings is formed by seven different but related β-subunits. Among them, only β1, β2, and β5 subunits and their corresponding immunosubunits β1i, β2i, and β5i are proteolytically active^9^, which have been described as possessing caspase-like, trypsin-like and chymotrypsin-like activities, respectively^10^,^11^. UPS plays an important role in regulating vascular diseases^12^. Many therapeutic studies of proteasome inhibition have been demonstrated in animal models with inflammation, including rheumatoid arthritis, asthma, and multiple sclerosis^11^,^13^,^14^. Studies also suggest that proteasome inhibitors could be used as immunosuppressive agents to treat deregulated and unwanted T-cell-mediated immune responses^11^,^13^,^14^. Particularly, treatment with low-dose PIs exerted favorable anti-inflammatory and anti-oxidative effects in vascular cells and a hypertensive rat model^12^,^15^,^16^ and atherosclerotic mice model^17^. Bortezomib (BTZ, also known as PS341, velcade) is a new and selective proteasome inhibitor for the 20S proteasome chymotryptic activity with limited activity on other enzymes^8^,^11^. However, little is unknown whether low-dose BTZ can effectively modulate AAA formation.

In this study, we sought to investigate the effect of proteasome activation on AAA formation in Apo E^−/−^ mice induced by chronic subcutaneous infusion of angiotensin II (Ang II)^18^. We also tested the potential therapeutic utility of BTZ in AAA *in vivo*. We found that proteasome activation significantly promoted inflammation and phenotypic SMC switching. In contrast, treatment of mice with low-dose BTZ markedly reversed these effects and Ang II-induced AAA formation. Thus, these results suggest that proteasome activation plays a critical role in the development of AAA via multiple mechanisms. The proteasome inhibitor BTZ may be a novel therapeutic drug for the treatment of AAA disease.

## Results

### Proteasome Activity and Subunits are upregulated in AAA Ttissues from Human and Apo E^−/−^ Mice

**Figure 1:**
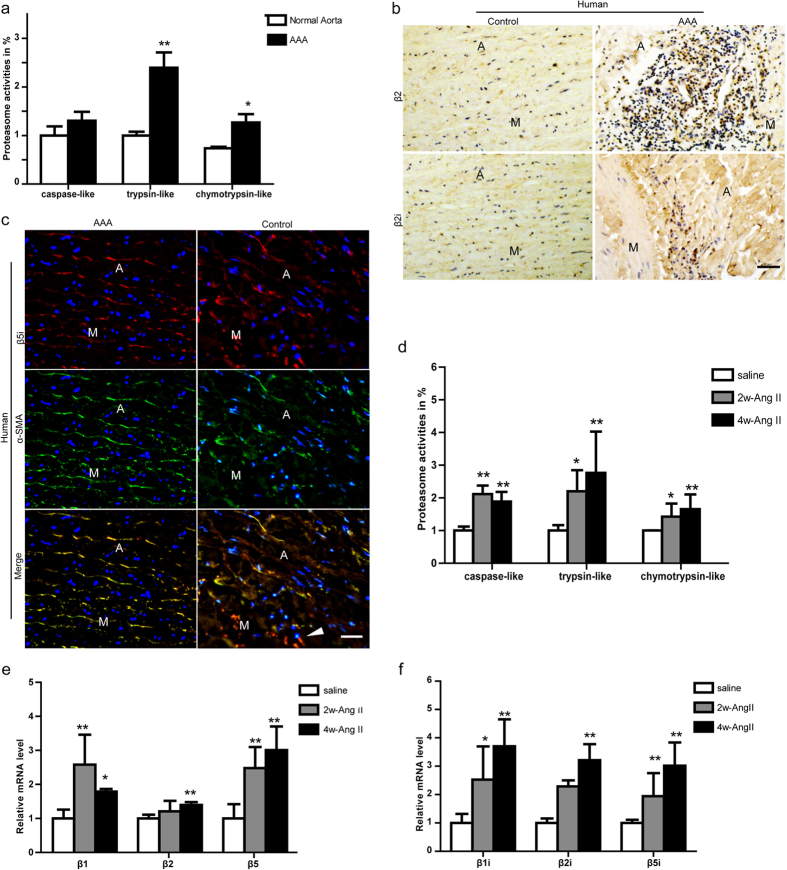
The proteasome is activated in the abdominal aorta of human AAA tissue or Ang II-infused apo E^−/−^ mice. (**a**) The proteasome activities including caspase-like, trypsin-like, and chymotrypsin-like activities were measured in aortic tissue from human. Activities are indicated as percentages of those in control aorta. (**b**) The expression of β2 and β2i was examined by immunohistochemistry. (**c**) The expression of β5i (red) and α-smooth muscle-actin (α-SMA) (green) was detected by double immunostaining. Nuclei were counterstained with DAPI (blue). (**d**) The proteasome activities were measured during the development of AAA in Apo E^−/−^ mice. (**e**,**f**) The qPCR analysis for the mRNA expression of β1, β2, β5, β1i, β2i and β5i during the development of AAA in Apo E^−/−^ mice. GAPDH was used as an internal control. Data expressed as means ± S.E.M. (n = 6 per group). *P < 0.05 vs Saline; ** *P* < 0.01 vs Saline. Scale bars: 50 μm. M: media; A: adventitia.

Previous studies have shown that UPS activation plays an important role in regulating vascular inflammation and SM marker gene expression^19^. Therefore, we first determine the proteasome activities in the abdominal aorta of human AAA tissue. As shown in Fig. 1a, proteasome peptidase activities, including the trypsin-like (of β2/β2i) and chymotrypsin-like (β1i/β5/β5i) were markedly increased in AAA tissue compared with age-matched control. Conversely, caspase-like activity (β1) was not markedly altered. Further, the increased expressions of β2/β2i and β5i subunits in the human AAA tissue were confirmed by immunohistochemistry or immunofluorescent staining (Fig. 1b,c). Particularly, several catalytic subunits such as β2, β2i, and β5i expression appeared to be co-localized areas of inflammation or with α-SMA (a marker for smooth muscle cells) in the AAA tissue (Fig. 1b,c).

To determine whether the activities of proteasome are altered during AAA formation and progression, we collected abdominal aortas in Apo E^−/−^ mice on the 14^th^ (2w-Ang II) and 28^th^ (4w-Ang II) day in Apo E^−/−^ mice after saline or Ang II infusion and measured 20S proteasome peptidase activities. As shown in Fig. 1d, the peptidase activities, including caspase-like (β1), trypsin-like (β2/β2i), and chymotrypsin-like (β1i/β5/β5i) in the Ang II-infused mice were significantly higher than the control (saline) on the 14^th^ or 28^th^ days. Since changes in proteasome activities in response to cellular stimuli are generally preceded by corresponding alterations in proteasome subunit expression, we then examined the expressions of proteasome subunits in the aortas of Ang II-infused mice. qPCR analysis showed that the mRNA levels of the constitutive catalytic subunits (β1, β2, and β5) and immunoproteasome catalytic subunits (β1i, β2i, and β5i) were highly upregulated in the abdominal aortas of Ang II-infused Apo E^−/−^ mice compared with the control (saline) on the 14^th^ or 28^th^ day (Fig. 1e,f). Together, these results suggest that proteasome activities (especially the trypsin-like and chymotrypsin-like) are significantly increased during AAA formation and may play an important role in this process.

### Inhibition of proteasome activity by BTZ reduces Ang II-induced AAA formation in Apo E^−/−^ Mice

**Figure 2:**
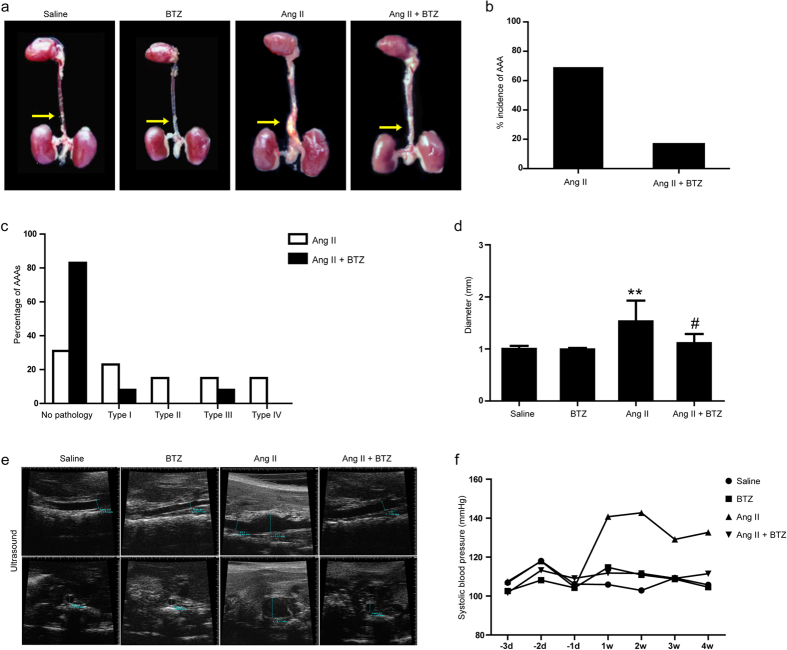
Proteasome inhibitor BTZ reduces the incidence and severity of abdominal aortic aneurysms in Ang II-infused apoE^−/−^ mice. The mice were injected with BTZ twice per week from the day Ang II infusion started. (**a**) Representative aortas. Arrows indicate aortic aneurysms. (**b**) Incidence of aortic aneurysm and dissection in animals (percentage). (**c**) Classification of aortic aneurysms in mice treated by Ang II or Ang II+BTZ as described by Daugherty. (**d**) Aortic diameter. (**e**) Representative images by high-frequency ultrasound of abdominal aortas. Longitudinal (top) and transverse (bottom) images were taken at the level of the suprarenal aorta. (**f**) Systolic blood pressure was measured in conscious mice every week by a computerized tail-cuff system. Data expressed as mean ± S.E.M. (n = 10 for Saline or BZT group, n = 15 for Ang II or Ang II+BTZ) **P < 0.01 vs saline, ^#^P < 0.05 vs Ang II.

We next determined whether inhibiting 20S proteasome affects AAA incidence in the Ang II-induced AAA mouse model. Previous study demonstrated that low-dose proteasome inhibitor BTZ (50 μg/kg body weight) attenuated atherosclerotic lesion development in low-density lipoprotein receptor–deficient mice with low mortality rate^17^. Thus, we used BTZ at a nontoxic dose (50 μg/kg, 2 times per week) to partially inhibit proteasome activity in Apo E^−/−^ mice treated with AngII or saline for 28 days^18^. We found that seven out of ten (70%) Ang II-treated mice developed suprarenal aortic aneurysms (Fig. 2a,b), and this Ang II effect was markedly attenuated in Ang II+BTZ group, where 2 out of 10 (20%) developed aneurysms (Fig. 2a,b). Further, based on the classification system by Daugherty *et al*.^20^, aneurysms induced by Ang II alone were more severe than those in the Ang II + BTZ group (Fig. 2c). The distribution of aneurysms among no aneurysms (none), Type I, Type II, Type III, and Type IV was significantly different between two groups (Fig. 2c). Additionally, Ang II-infused Apo E^−/−^ mice had a significantly larger suprarenal aortic diameter compared to the saline control group (1.50 ± 0.40 mm vs. 1.00 ± 0.06 mm). In contrast, Ang II + BTZ mice showed a markedly smaller aortic diameter than that in Ang II alone (1.20 ± 0.11 mm vs. 1.50 ± 0.40 mm) (Fig. 2d). Treatment with BTZ alone did not affect the aortic diameter (Fig. 2d). These results were further confirmed by high-frequency ultrasound (Fig. 2e). In addition, blood pressure increased persistently during Ang II treatment, which was markedly reversed by BTZ injection (Fig. 2f). Moreover, the survival rate up to 4 weeks was higher in Ang II+BTZ mice than in Ang II mice (83.3% vs. 66.7%) (Supplemental Fig. 1). There were no deaths in Saline and BTZ groups (Supplemental Fig. 1). Together, these results indicate that low-dose BTZ treatment can markedly attenuate Ang II-induced aneurysm incidence and severity.

### Low-dose BTZ inhibits Ang II-induced aortic wall remodeling in Apo E^−/−^ Mice

**Figure 3:**
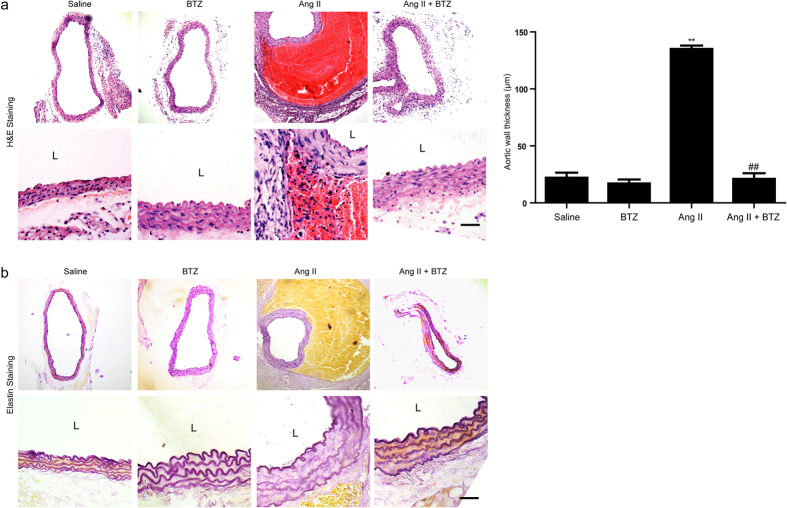
Proteasome inhibitor BTZ suppresses the aortic wall remodeling in Ang II-infused apoE^−/−^ mice. (**a**) The representation of H&E staining in abdominal aorta from Apo E^−/−^ mice (left). Bar graph shows the quantification of aortic wall thickness (right; n = 3 per group). (**b**) The representation of elastin staining in abdominal aorta from Apo E^−/−^ mice. Magnification:×100. Scale bar: 50 μm. Data expressed as mean ± S.E.M. **P < 0.01 vs Saline, ^##^*P* < 0.05 vs Ang II. L: lumen.

It is reported that artery wall remodeling is a hallmark of AAA formation and progression. Therefore, we determined the effect of proteasome activation on suprarenal artery remodeling in Apo E^−/−^ mice using H&E and VVG staining. As shown in Fig. 3a,b, Ang II infusion resulted in the abdominal aortic wall thickening, media disruption with thrombus formation, and elastic fiber breakage. However, these histological alterations were markedly suppressed in Ang II+BTZ group (Fig. 3a,b). There was no significant difference in the aortic wall remodeling alterations between saline and BTZ group (Fig. 3a,b).

### Low-dose BTZ suppresses Ang II-induced MMP activity in Apo E^−/−^ mice

**Figure 4:**
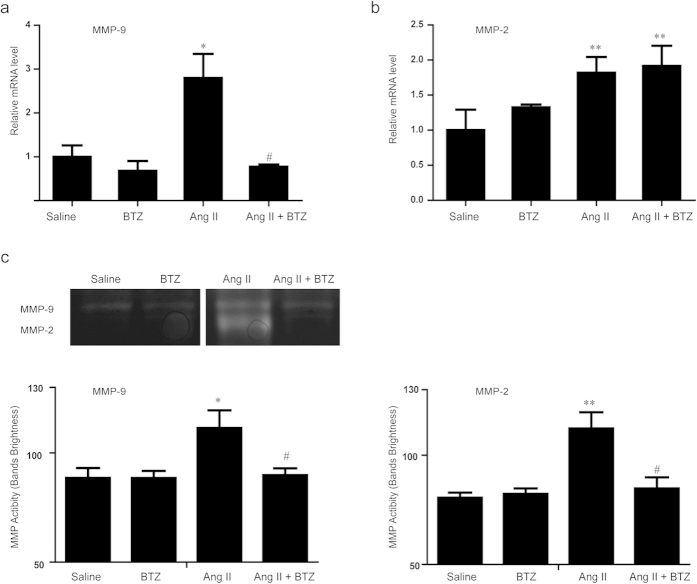
Proteasome inhibitor BTZ decreases MMP activity in Ang II-infused apoE^−/−^ mice. (**a**,**b**) The qPCR analysis for the mRNA expression of MMP-2 and MMP-9 in abdominal aorta from four groups. GAPDH was used as an internal control. (**c**) The representation of MMP zymography gel (the upper panel). The analysis for the brightness of the bands (the lower panel). (n = 4 per group). Data expressed as mean ± S.E.M. *P < 0.05 vs Saline, **P < 0.01 vs saline, ^#^*P* < 0.05 vs Ang II.

Since Ang II markedly increased the expression and activity of matrix metalloproteases (MMPs)^21^, which play a critical role in AAA formation by disrupting the ECM proteins^21^,^22^, we then detected aortic MMP-2 and MMP-9 expressions in saline- and Ang II–infused Apo E^−/−^ mice. qPCR analysis showed that Ang II infusion for 4 weeks significantly up-regulated mRNA expressions of MMP-2 and MMP-9 in Apo E^−/−^ mice, whereas, BTZ significantly reduced only MMP-9 expression rather than MMP-2 (Fig. 4a,b). To further test whether BTZ affected MMP-2 and MMP-9 activity, we also performed gelatin zymography. Ang II infusion in Apo E^−/−^ mice significantly increased MMP-2 and MMP-9 activity compared with control mice, and their activity was markedly attenuated by BTZ injection (especially the MMP-2 activity) (Fig. 4c). BTZ did not significantly influence MMP-2 and MMP-9 expression or activity under saline infusion (Fig. 4a–c).

### Low-dose BTZ inhibits Ang II-induced inflammatory response in Apo E^−/−^ mice

**Figure 5:**
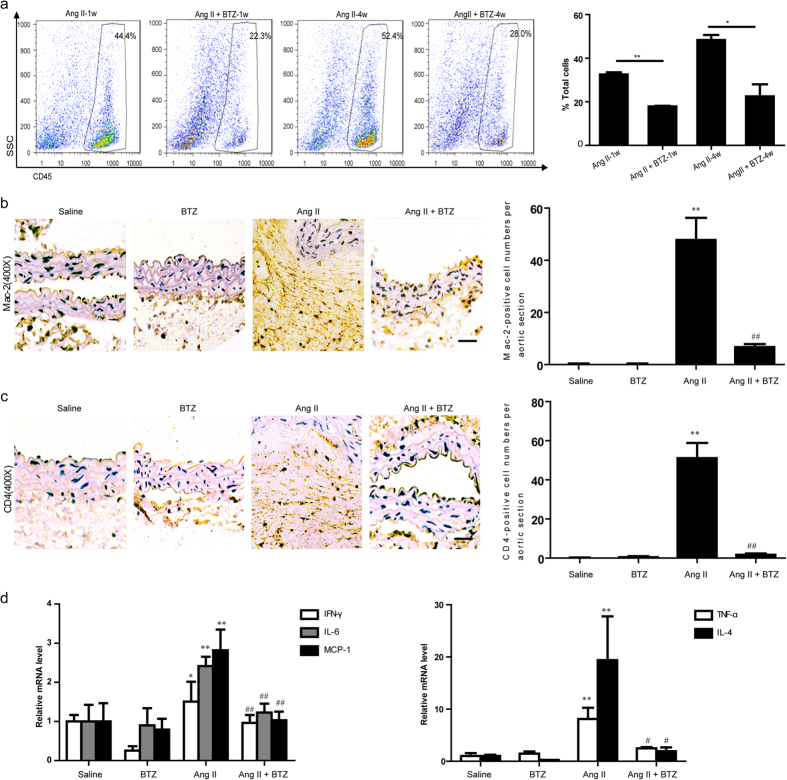
Proteasome inhibitor BTZ inhibits the accumulation of immune cells and the expression of inflammatory cytokines in Ang II-infused apoE^−/−^ mice. (**a**) The representation of flow-cytometry analysis for quantifying the number of CD45^+^ immune cells in in abdominal aorta (left). Bar graph shows the percentage of CD45^+^ immune cells (right; n = 3 per group). (**b**) and (**c**) The representation of immunohistochemical staining for Mac-2 and CD4^+^ T cells in abdominal aorta from Apo E^−/−^ mice (left). Bar graph shows the quantification of the percentage of Mac-2 and CD4^+^ T cell areas (right; n = 3 per group). (**d**) qPCR analysis for the mRNA expression of TNF-α, IFN-γ, IL-4, IL-6 and MCP-1 in abdominal aorta. GAPDH was used as an internal control. Magnification:×100. Scale bar: 50 μm. Data expressed as mean ± S.E.M. *P < 0.05, **P < 0.01 vs saline. ^#^*P* < 0.05, ^##^*P* < 0.01 vs Ang II.

Inflammation has been reported to play a critical role in AAA formation^23^. To determine whether inhibiting proteasome activity *in vivo* was efficient in decreasing immune cell infiltration into the aorta, we measured the number of CD45^+^ hematopoietic cells in harvested aorta by flow cytometry. BTZ injection significantly decreased the proportion of the infiltrated immune cells induced by Ang II at week 1 or 4 (Fig. 5a). To further clarify which immune cells were reduced by BTZ, we detected the accumulation of macrophages and CD4^+^ T cells, which were reported to be the main cells to play a key role in AAA formation. Immunohistochemical staining demonstrated that the numbers of Mac-2-positive macrophages and CD4^+^ T lymphocytes were significantly increased in Ang II–infused mice, and the increased cells were markedly attenuated in Ang II+BTZ group (Fig. 5b,c). Further, Ang II–induced expressions of macrophage- or T-cell-specific cytokines, such as TNF-α, IFN-γ, IL-4, IL-6, and MCP-1, were also markedly suppressed in the abdominal aorta by BTZ injection (Fig. 5d). BTZ did not affect inflammatory cells and other cytokines under saline infusion (Fig. 5d). Collectively, these results suggest that partial proteasome inhibition abrogates the proinflammatory effects in aorta caused by Ang II infusion.

### Low-dose BTZ inhibits AngII-induced proliferation but not apoptosis of aortic smooth muscle cells in Apo E^−/−^ mice

**Figure 6:**
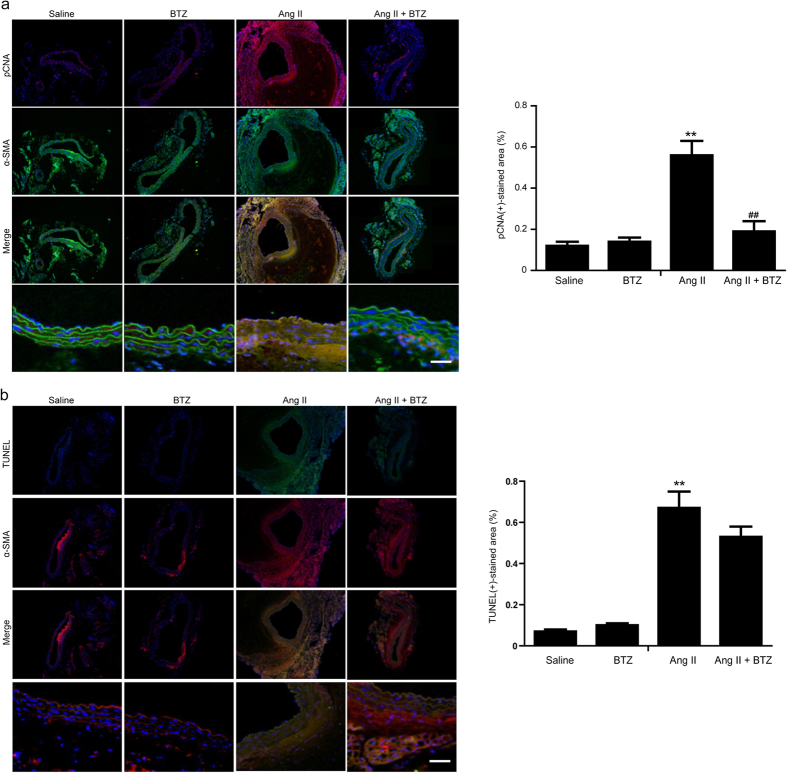
Proteasome inhibitor BTZ attenuates the proliferation of the aortic smooth muscle cells in Ang II-infused apoE^−/−^ mice. (**a**) pCNA (green) and α-SMA (red) double staining to detect proliferative SMCs in abdominal aorta from Apo E^−/−^ mice (left). Nuclei were counterstained with DAPI (blue). Bar graph shows the percentage of pCNA positive areas (right, n = 3 per group). (**b**) TUNEL (green) and α-SMA (red) double staining to detect apoptotic SMCs (left). Bar graph shows the percentage of TUNEL positive areas (right, n = 3 per group). Data expressed as mean ± S.E.M. **P < 0.01 vs saline. ^##^*P* < 0.01 vs Ang II.

Aortic smooth muscle cell (SMC) proliferation and apoptosis are important mechanisms for AAA formation^6^,^24^, we then determined the effect of partial proteasome inhibition on the aortic SMCs by immunostaining. Consistent with previous study^25^, pCNA staining was co-localized with α-SMA positive cells and was stronger in the adventitia and media of Ang II-infused mice compared with saline-treated mice (Fig. 6a), indicating Ang II infusion increases SMC proliferation in the aorta. Conversely, these above effects were significantly abolished in the Ang II+BTZ group. However, BTZ treatment alone had no effect on SMC proliferation as determined by pCNA staining (Fig. 6a). Further, we tested the effect of partial proteasome inhibition on the SMC apoptosis by TUNEL assay. As shown in Fig. 6b, Ang II infusion markedly increased TUNEL-positive cells mainly within the α-SMA-positive SMCs compared with saline group. However, low-dose BTZ treatment did not influence SMC apoptosis under basal condition or Ang II infusion (Fig. 6b). These results suggest that low-dose BTZ treatment inhibits Ang II-induced AAA formation in Apo E^−/−^ mice, probably by changing the proliferative state.

### Low-dose BTZ reversed the phenotypic switching of SMCs in AAA induced by Ang II

**Figure 7:**
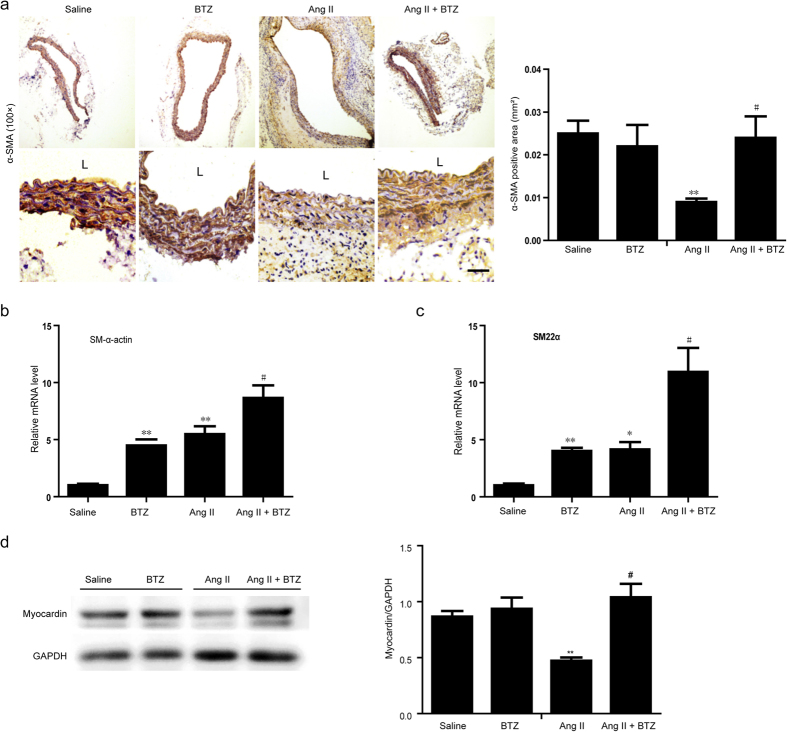
Proteasome inhibitor BTZ reverses the phenotypic switching of the aortic smooth muslce cells in Ang II-infused apoE^−/−^ mice. (**a**) The representation of immunohistochemical staining for α-SMA in abdominal aorta from from Apo E^−/−^ mice (left). Bar graph shows the quantification of the percentage of α-SMA positive cell areas (right; n = 3 per group). (**b**) qPCR analysis for the mRNA expression of SM α-actin and SM22αin abdominal aorta (n = 4 per group). (**d**) Western Blot analysis for the protein levels of myocardin in abdominal aorta (left). Bar graph shows the quantification relative to GAPDH level (right; n = 3 per group). Data expressed as mean ± S.E.M. *P < 0.05, **P < 0.01 vs saline. ^#^*P* < 0.05 vs Ang II.

Phenotypic SMC modulation plays a central role during AAA formation^24^, and above data (Fig. 6a) indicated that low-dose BTZ inhibited the Ang II-induced proliferative state of aortic SMCs in Apo E^−/−^ mice. We then detected the phenotype marker expressions in abdominal aortas in each group at 28 days after Ang II infusion. Immunohistochemistry demonstrated that the α-SMA expression was markedly decreased in Ang II group compared with saline or BTZ group, whereas this change was markedly reversed by low-dose BTZ injection (Fig. 7a). qPCR analysis further revealed that both BTZ and Ang II significantly increased SM α-actin and SM22α mRNA expressions compared with saline group at 28 days, and these effects were further enhanced in the Ang II+BTZ group (Fig. 7b,c). Myocardin, a serum response factor (SRF) coactivator, is recently thought to play a critical role in differentiating vascular smooth muscle cells (SMCs). Therefore, we measured the myocardin expression in the abdominal aortas of Apo E^−/−^ mice. Western blot analysis showed a low myocardin expression level in the Ang II group compared with the saline or BTZ alone under basal condition. However, the decreased myocardin protein level was markedly reversed in Ang II+BTZ group (Fig. 7d). There was no significant difference in the myocardin protein level in the AAAs between groups after saline infusion (Fig. 7d). Taken together, these results demonstrate that BTZ can inhibit myocardin degradation, and the increased myocardin cause more SM marker expression.

## Discussion

Here we for the first time demonstrated that activities and expressions of proteasome subunits were significantly upregulated in AAA tissue from both human patients and Ang II-infused Apo E^−/−^ mice. Treatment of mice with low-dose proteasome inhibitor BTZ markedly attenuated proteasome activities and Ang II-induced AAA formation. Further, low-dose BTZ treatment markedly reduced Ang II-induced proinflammatory response and inhibited MMP activities, but simutaneously reversed the expression of differentiated markers and myocardin in AAA tissue. Thus, these data provide the novel *in vivo* evidence for the critical role of proteasome activation in promoting AAA formation in ApoE^−/−^ mice after Ang II infusion.

Proteasomes are large intracellular protein complexes that specialize in degrading cellular proteins that are either unneeded or damaged^26^. They exert the central role in a number of physiological and pathophysiological processes, including oxidative stress, inflammation, and SMC differentiation and apoptosis^7^. A recent study found that 20S proteasome proteolytic activity was unimpaired, and ubiquitin conjugates were accumulated in the coronary arteries of pigs received a high-cholesterol diet^27^. The pathological process of such a disease is similar to AAA, including inflammatory cell infiltration, vascular cell apoptosis, and increased collagen degradation^28^. Importantly, we previously found that UPS also plays a key role in the physiological control of SMC phenotype and vessel tone, which may be an important implication for atherosclerosis and AAA^29^. Recently, various inhibitors for proteasome activation have been developed^30^. Among these, BTZ is a selective and reversible proteasome inhibitor for the 20S proteasome chymotryptic activity^8^,^11^, and has shown cytotoxicity against a broad range of human tumor cells^8^,^11^. Notably, low-dose proteasome inhibitor has anti-oxidative and anti-inflammatory effects and inhibits atherosclerotic lesion formation^18^. However, the exact mechanism for proteasome to promote AAA formation remains unclear. Similarly, in this study, low-dose BTZ injection significantly reduced AAA incidence and severity by attenuating the local inflammation responses of abdominal aorta resulting in reduced MMP activities, elastin degradation, and SMC phenotypic change induced by AngII infusion (Figs 2, 3, 4, 5, 6, 7). Together, these results demonstrate that proteasome activation plays a critical role in the development of AAA through multiple mechanisms.

Interestingly, we observed that the blood pressure in Ang II group was decreased after 2 weeks of Ang II infusion (Fig. 2f). In fact, chronic Ang II infusion was reported to cause vascular injury, including endothelial damage, wall thickness, elastin break and inflammation leading to impairment in vascular reactivity and endothelial function, and decreased blood pressure^31^,^32^,^33^. The development of AAA has been considered as an autoimmune disease because it shares many characteristics, such as genetic predisposition, organ specificity, and chronic inflammation. Inflammatory cells accumulate in AAA lesions with predominantly macrophages and CD4^+^ T cells (3- to 20-fold greater than CD8^+^ T cells)^34^. Thus, macrophages and T cells may play a major role in the pathogenesis of AAA disease^35^,^36^. Recently, more evidence also supports the idea that Th2 type cytokines and chemokines localize in the human AAA disease’s late stages^35^, and Th2 predominantly contributes to AAA formation^37^. Thus, inhibition of inflammatory cell infiltration has become a therapeutic target for the treatment of AAA formation. Interestingly, proteasomal activity is required for the essential immune functions of activated CD4^+^ T cells, and could be defined as a potential target for the suppression of deregulated T cell-mediated immune responses^38^. Consistent with the previous findings^22^, Studies also suggest that proteasome inhibitors could be used as immunosuppressive agentsintraperitoneal BTZ injection markedly inhibited the accumulation of proinflammatory cells, including Mac-2-macrophages and CD4^+^ T cells in the aneurysm wall induced by Ang II infusion (Fig. 5a–c). Moreover, low-dose BTZ injection mainly prevented Th2 cytokine expression, including IFN-γ, TNF-α, IL-4, IL-6 and MCP-1 in Ang II-induced AAA, presumably by blocking NF-κB signaling pathway^39^ (Fig. 5d). Together, activation of proteasome mediates AAA formation partially via promoting accumulation and activation of macrophages and CD4^+^ T cells.

It has been reported that activated inflammatory cells markedly secret proteases that regulate ECM protein turnover. Among the proteases, MMPs, especially MMP-2 and MMP-9, are considered as the major proteases in the aortic wall destruction process^29^. Therefore, we chose MMP-2 and MMP-9 to test whether proteasome plays an important role in ECM degradation. Here our data showed that the mRNA expressions and activities of MMP-2 and MMP-9 were both increased in the Ang II-infused aorta, but only MMP-9 was significantly reduced by low-dose BTZ (Fig. 4a,b). However, both MMP-2 and MMP-9 activities were markedly abolished by low-dose BTZ (especially the MMP-2 activity) (Fig. 4c). Previous results confirmed that in human AAA tissue, strong MMP-9-positive staining was detected in all lymphocytes and mast cells without expressing MMP-2. However, strong expressions of MMP-2 and MMP-9 were observed in all macrophages within AAA. Thus, we suppose that the divergence of BTZ’s effectiveness on the proliferation of macrophages and T lymphocytes may contribute to the difference between MMP-2 and MMP-9 gene expression.

The phenotypic modulation of SMCs has been well established in various vascular diseases including atherosclerosis and hypertension^29^. In the murine carotid artery injury model, the phenotypic SMC change is observed, and SM marker genes including SM22α and SM-α actin are reduced, which is accompanied by SMC proliferation and migration^40^. It was recently demonstrated that phenotypic SMC modulation plays an important role during AAA formation^24^. In this study, we found low-dose BTZ inhibited Ang II-induced SMC proliferation and reversed SMC differentiated marker expression, but did not affect aortic SMC apoptosis (Figs 6 and 7). In fact, BTZ has never been seen as an effective anti-apoptotic molecule. In contrast, BTZ exerts a pro-apoptotic role in most cases^8^. Furthermore, as our previous study described, ubiquitylation and degradation of myocardin also regulate SMC phenotypic change^29^. Therefore, we analyzed myocardin expression levels to further elucidate the exact mechanism of BTZ’s modulation effect in the phenotype switching. Our results indicate that low-dose BTZ can markedly inhibit the myocardin degradation leading to enhanced SM marker gene expression (Fig. 7b–d).

In conclusion, to our best knowledge, we have been first to demonstrate that proteasome activation plays a critical role in promoting AAA formation *in vivo*. This was associated with enhanced inflammatory cell infiltration, MMP activity and the SMC phenotype switch in Ang II-induced AAA tissues. We also demonstrated that low-dose proteasome inhibitor BTZ prevented these effects *in vivo*. These findings not only further highlight proteasome’s involvement in AAA pathophysiology, but also suggest BTZ might be a new therapeutic target for treatment of AAA formation. Further investigations in other animal models of AAA are needed to verify the clinical use of BTZ as a pharmacological therapy.

## Methods

### Ethics statement

All animal studies were approved by the Institutional Animal Care and Use Committee of Peking Union Medical College Hospital, and experiments conformed to the Guide for the Care and Use of Laboratory Animals (National Institutes of Health publication No.85–23,1996).

### Antibodies and reagents

Antibodies to β2i (ab77735), β2 (ab22650), and α-smooth muscle actin (α-SMA) (ab5694) were purchased from Abcam Inc. Antibodies to Mac-2 (sc-20157), myocardin (sc-33766), GAPDH (sc-365062) and 4′,6-diamidino-2-phenylindole (DAPI) were from Santa Cruz Biotechnology. Antibodies to CD4 (NBP1-19371) were from Novus. All antibodies were used in a 1:200 dilution. For immunofluorescent staining, antibodies to β5i (ab3329), pCNA (ab18197) and α-SMA were from Abcam Inc. Anti-mouse or anti-rabbit conjugated antibodies were from Cell Signaling Technology. TUNEL assay kit was from Promega. For Flow Cytometry, antibodies to CD45 (557659) was from BD Bioscience Pharmingen. Proteasome inhibitor bortezomib (BZT, sc-217785A) was purchased from Santa Cruz Biotechnology. Ang II (A9525) was from Sigma.

### Animals and treatment

Male C57BL/6J and Apo E^−/−^ mice (backcrossed 10 times into the C57BL/6J background) were obtained from Department of Laboratory Animal Science (Peking University Health Science Center, Beijing, China). All mice were bred as littermate controls, and housed in a pathogen-free barrier facility. Male mice (10 weeks of age) were implanted with Alzet osmotic minipumps (Model 2004, Durect Corporation), filled either with saline vehicle or Ang II solutions (1,000 ng/kg/min) up to 4 weeks^18^. Aortas were harvested from mice at 1, 2 and 4 weeks after Ang II infusion. For drug administration assay, bortezomib (BZT) was dissolved in dexamethasone (1 μl for each mouse each time) firstly and mixed with 0.9% saline with a final volume of 100 μl before injection intraperitoneally into a mouse. The mice were divided into four groups: **Saline**, **BZT** (50 μg/kg, 2 times per week), **Ang II**, and **AngII+BZT** (50 μg/kg, 2 times per week). All mice were anesthetized. The aortic tissues were removed and prepared for further histological and molecular analysis.

### Patients and sample processing

Abdominal aortic tissues and blood were obtained from patients with AAA patients and control who underwent surgical procedures as described previously^1^,^2^. The control subjects without AAA were obtained from heart transplant donors, who have similar clinical characteristics, including age, sex, ethnic background, etc (Supplemental table 1). Abdominal aortic aneurysms were confirmed by aortic morphological analysis as described previously^1^,^2^. Abdominal aortic tissues were either preserved at −80 °C for proteasome peptidase activity assay or fixed in 4% paraformaldehyde for immunohistochemistry and immunofluorescence staining. The study protocol was approved by the Ethical Committee of Peking Union Medical College Hospital, and informed consent was obtained from the individuals. All experiments were performed in accordance with the relevant guidelines and regulations.

### Noninvasive blood pressure measurement

Blood pressure and heart rate were measured noninvasively using the Softron BP-98A Blood Pressure Analysis System (Softron Systems, Tokyo, Japan) as described previously^1^,^2^. To ensure accuracy and reproducibility, the mice were trained for 3 days prior to the experiment, and measurements were taken each day at the same time.

### Analysis and quantification of AAA

To quantify the incidence and size of AAA, abdominal aortic diameter was measured with a Vevo 770 ultrasound system (VisualSonics Inc.) and MNI, respectively, as described^1^,^2^. The out diameter of the suprarenal aorta was also measured with a caliper. For quantifying aneurysm incidence, an aneurysm was defined as a 50% or greater increase in the external width of the suprarenal aorta compared to aortas from saline-infused mice as described previously^1^,^2^. Aneurysm severity was rated from Type I to Type IV according to the method of Daugherty *et al*.^20^.

### Histology, immunohistochemical staining and TUNEL assay

All mice were killed after 4 weeks of treatment. For morphological analysis, aortas were perfused with normal saline and fixed with 10% PBS and formalin. Whole aortas were harvested and aortic tissue was removed from the ascending aorta to the ileac bifurcation. After fixed for 24 h and embedded in paraffin. Aortic cross-sections (5 μm) were prepared. Paraffin sections were stained with H&E as described^20^. Immunohistochemical staining was performed with primary antibodies, including β2, β2i, Mac-2, CD4 and α-SMA, β5i and pCNA. TUNEL assay was performed according to manufacturer’s instructions as described^1^,^2^. Digital photographs were taken at 10× or 20× magnification of over 10 random fields from each aorta, and the positive areas were calculated by Image Pro Plus 3.0 (Nikon, Tokyo, Japan).

### Proteasome activity assay

Proteasome activities in the aorta tissues were performed using fluorogenic peptide substrates as described^41^,^42^,^43^. Briefly, abdominal aorta samples were homogenized at 4 °C in 10 volumes HEPES buffer (50 mM, pH 7.5) containing: KCl 20 mM, MgCl25 mM, DTT 1 mM. Cell debris was removed by centrifugation for 15 minutes at 12,000 g, and the supernatants were immediately used for protein concentration assay. The caspase like activity was determined with Z-LLE-AMC (45 μmol/L), trypsin-like activity with Ac-RLR-AMC (40 μmol/L), and chymotrypsin-like activity with Suc-LLVY-AMC (18 μmol/L) as substrates in the absence or presence of a proteasome inhibitor, MG-132 (20 μmol/L) or epoxomycin (5 μmol/L). 5 μg of protein extract is added to 200 μl of the HEPES buffer containing the fluorogenic substrates and incubated at 37 °C for 1 h. The fluorescence intensity was measured using a Perkin Elmer 2030 Multilabel Microplate Reader with the excitation at 380 nm and emission at 460 nm.

### RNA isolation and quantitative real-time PCR analysis

Total RNA was extracted from the fresh abdominal aortas using Trizol method(Invitrogen, Carlsbad, CA) according to the manufacturer’s instructions as described^41^. The first strand cDNA was synthesized from 1–2 μg of total RNA by oligo (dT)-primed RT (iScript cDNA synthesis kit; Bio-Rad Laboratories). All standards and samples were assayed in triplicate. All samples were normalized to the relative levels of GAPDH and results were expressed as fold increase in relative levels. Primers were designed using PrimerExpress (Applied Biosystems) software as follows, see Table 1.

### Flow cytometry

Aortic tissue from mice was minced and incubated for 1 hour at 37 °C with aorta dissociation enzyme stock solution which was prepared as described^1^,^2^,^44^,^45^,^46^. The cell suspension was made for flow cytometry analysis in FACS buffer (PBS supplemented with 1% BSA and 0.05% NaN3). The cell surface was labeled with the antibody APC-Cy7–labeled CD45 (BD Biosciences). FACS data were analyzed using FlowJo software.

### Western blot analysis

Tissue lysis and Western blot analysis were performed as described previously^1^,^2^. Aortas were lysed in extraction buffer containing in mmol/L: Tris/HCl 50 (pH 7.4), KCl 154, glucose 5, EDTA 0.5, PMSF 1, DTT 2, and 1% Triton X-100. Total protein (20 μg per lane) was subjected to SDS-PAGE and membranes were probed with the respective antibodies: anti-myocardin (1:200), and GAPDH (1:1000).

### MMP activity Assay

The activities of MMP2 and MMP9 in aortas from mice were evaluated according to the protocols as described^1^,^2^. Five micrograms of protein from homogenates of aortic tissues was eletrophoresed in SDS-PAGE gels containing 1 mg/ml gelatin. Then the gel was washed in 2.5% Triton X-100 and incubated in zymography developing buffer. Finally, the gel was stained with Coomassie brilliant blue.

### Statistical analysis

Quantitative results are expressed as mean ± S.E.M. Comparisons of parameters between two groups were made by *t* test. Comparisons of parameters among more than two groups were made by one-way analysis of variance, and comparisons of different parameters between each group were made by a post hoc analysis using a Bonferroni test. Kaplan-Meier survival curves were constructed and analyzed using log-rank test. Nonparametric Manne-Whitney U and Kruskal Wallis tests were performed when the sample size was smaller. Statistical significance was evaluated with SPSS13.0^44^. A value of P < 0.05 was considered to be statistically significant.

## Additional Information

**How to cite this article**: Ren, H. *et al*. Inhibition of Proteasome by Low-dose bortezomib Attenuates Angiotensin II-induced Abdominal Aortic Aneurysm in Apo E^−/−^ Mice. *Sci. Rep.* **5**, 15730; doi: 10.1038/srep15730 (2015).

## Supplementary Material

Supplementary Information

## Figures and Tables

**Table 1:** Primers for real-time PCR analysis.

Genes	Forward primers	Reverse primers
β1 subunit	5′-TAATTGGCTGCAGTGGTTTCC-3′	5′-AAGCGCCGTGAGTACAGGAT -3′
β2 subunit	5′-GATGAAGGACGATCATGACAAGAT-3′	5′-TGGGAGACAATTCATATCCATTCC -3′
β5 subunit	5′-CGCAGCAGCCTCCAAACT-3′	5′-GAAGGCGGTCCCAGAGATC-3′
β1i subunit	5′-TAGCTGACATGGCCGCCTA-3′	5′-TGGTCCCAGCCAGCTACTATG-3′
β2i subunit	5′GGAACCCACAGGAGGCTTCT-3′	5′-GTCCGCTCCCAGGATGACT-3′
β5i subunit	5′-AAGGATGAACAAAGTGATCGAGATT-3′	5′-TGCTGCAGACACGGAGATG-3′
MCP-1	5′-GACCCGTAAATCTGAAGCTAA -3′	5′-AAGACATCCTCACTGGTCACAC-3′
TNF-α	5′- GGCAGGTCTACTTTGGAGTCATTG-3′	5′- GTTAGAAGGACACAGACTGG-3′
INF-γ	5′-AAAGAGATAATCTGGCTCTGC-3′	5′-GCTCTGAGACAATGAACGCT-3′
TGF-β	5′- TGACGTCACTGGAGTTGTACGG-3′	5′- GGTTCATGTCATGGATGGTGC-3′
IL-4	5′-TCAACCCCCAGCTAGTTGTC-3′	5′-TGTTCTTCGTTGCTGTGAGG-3′
IL-6	5′-CCAGAAACCGCTATGAAGTTCCT-3′	5′-AGAACCCTGACTACGACCAC-3′
MMP-2	5′-CGCTCAGATCCGTGGTGA-3′	5′-TTCCTGGCCAAATAAACCGC-3′
MMP-9	5′-CAATCCTTGCAATGTGGATG-3′	5′-AGTAAGGAAGGGGCCCTGTA-3′
SM α-actin	5′-AATGCAGAAGGAGATCACGG-3′	5′-TCCTGTTTGCTGATCCACATC-3′
SM22α	5′-TCCAGACTGTTGACCTCTTTG-3′	5′-TCTTATGCTCCTGCGCTTTC-3′
GAPDH	5′-TGTACCGTCTAGCATATCTCCGAC-3′	5′-ATGATGTGCTCTAGCTCTGGGTG-3′
