# Supplementary material for: Inhibition of Proteasome Activity by Low-dose Bortezomib Attenuates Angiotensin II-induced Abdominal Aortic Aneurysm in Apo E−/− Mice
Source: Sci Rep. 2015 Oct 28;5:15730. doi: 10.1038/srep15730 (PMC4623715; doi:10.1038/srep15730)
Supplement: Supplementary Information [file srep15730-s1.pdf]

Inhibition of Proteasome Activity by Low-dose Bortezomib Attenuates Angiotensin II-induced Abdominal Aortic Aneurysm in Apo E$^{-/-}$ Mice

Hualiang Ren[1], Fangda Li[1], Cui Tian[2], Hao Nie[1], Lei Wang[3], Huihua Li[3,4]* & Yuehong Zheng[1]*

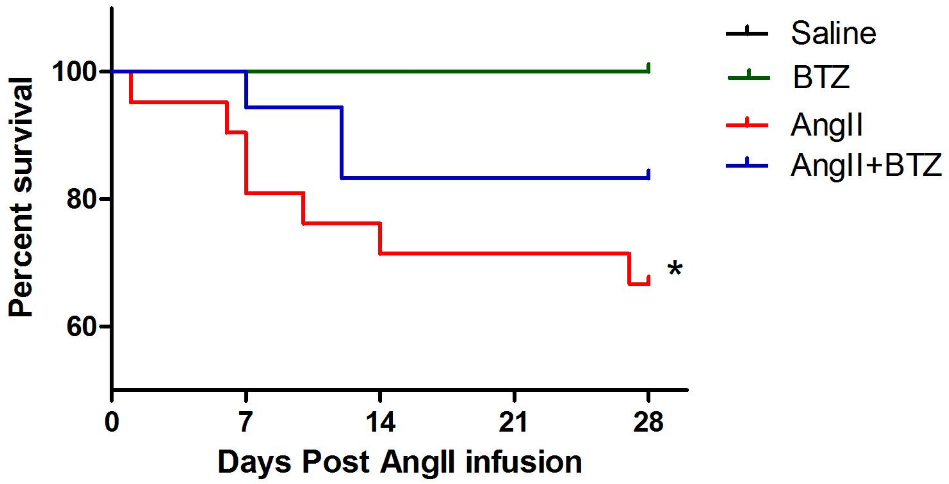

Supplemental figure 1│Inhibition of proteasome activity by BTZ reduces Ang II-induced AAA formation in Apo E$^{-/-}$ Mice. Kaplan-Meier survival curves in mice up to 4 weeks after Ang II with or without BTZ injection. (n=10-21 per group). *$P < 0.05$ vs. Saline.

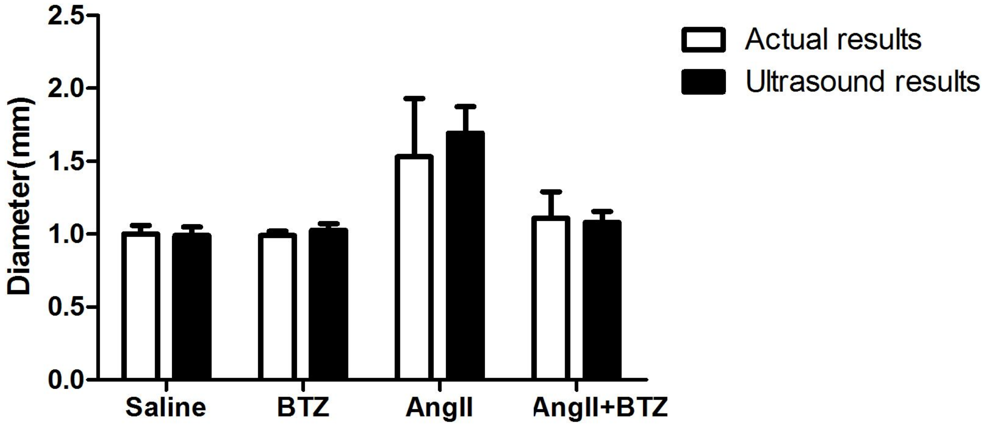

Supplemental figure 2│Inhibition of proteasome activity by BTZ reduces Ang II-induced AAA formation in Apo E$^{-/-}$ Mice. Bar graph shows the aortic diameter of ultrasound results with actual diameter size of AAA between groups.

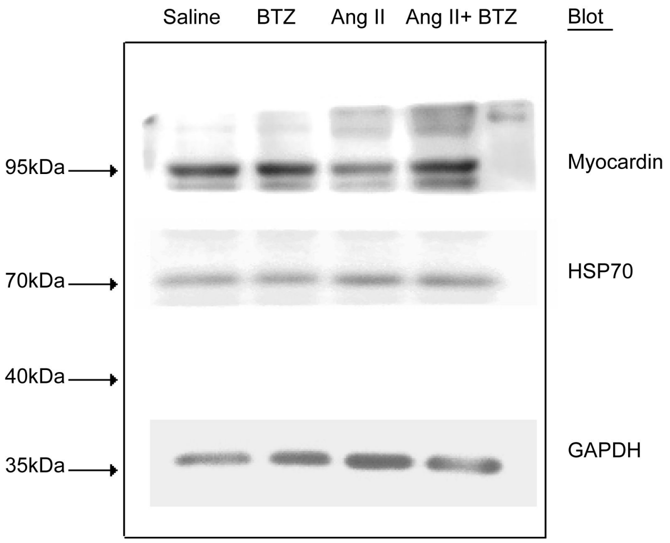

Supplemental figure 3｜Low-dose BTZ reversed the phenotypic switching of SMCs in AAA induced by Ang II. The original Western Blot film is shown.

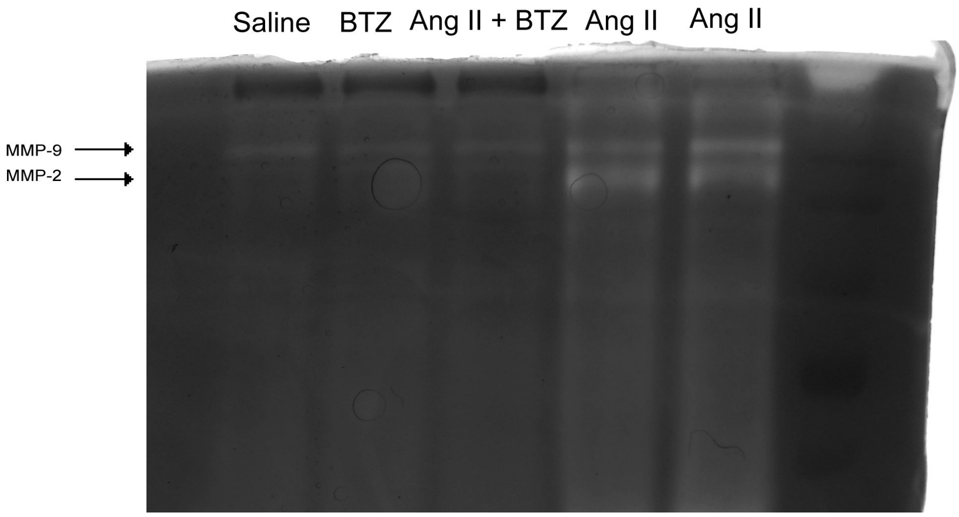

Supplemental figure 4｜Low-dose BTZ decreases MMP activity in Ang II-infused apoE$^{-/-}$ mice. The original Zymography film is shown.

Supplementary table 1. Demographic and clinical characteristics of subjects

	AAA<br>n=3	Control<br>n=3
Age, years (range)	65.1 (54-72)	65.5 (60-70)
Male sex	3	3
Carotid atherosclerosis	2	2
Smoking	1	2
Hypertension	3	3
Cerebrovascular disease	0	0
Diabetes mellitus	1	1
